# CT Imaging Before Extracorporeal Cardiopulmonary Resuscitation in Hypothermic Cardiac Arrest With Trauma

**DOI:** 10.7759/cureus.66629

**Published:** 2024-08-11

**Authors:** Kenshiro Wada, Ryuichiro Kakizaki, Yasuhiro Horio, Rina Oizumi, Kohei Kato

**Affiliations:** 1 Department of Emergency Medicine, Obihiro Kosei General Hospital, Obihiro, JPN; 2 Department of Emergency Medicine, Sapporo Medical University, Sapporo, JPN

**Keywords:** computed tomography, trauma, cardiac arrest, extracorporeal cardiopulmonary resuscitation, accidental hypothermia

## Abstract

Hypothermic cardiac arrest (HCA) with concomitant trauma presents a significant clinical management challenge. In these case reports, computed tomography (CT) imaging was performed before extracorporeal cardiopulmonary resuscitation (ECPR) in patients with cardiac arrest, accidental hypothermia, and trauma. The first case involved a 74-year-old male who collapsed outside his home under freezing conditions. Upon arrival at the emergency department (ED), he was in cardiac arrest with a core body temperature of 25.0°C and suspected head trauma. CT imaging revealed minor traumatic brain injuries and bilateral femoral fractures. ECPR was initiated after CT imaging, which led to successful rewarming and full neurological recovery. The second case describes a 32-year-old female who jumped from a bridge, experienced cardiac arrest during the rescue, and had a core temperature of 17.4°C. CT imaging before ECPR revealed no significant trauma. Despite prolonged resuscitation, the patient showed a complete neurological recovery. CT imaging before ECPR allows appropriate patient selection by ruling out cardiac arrest before hypothermia and major hemorrhagic complications. Hypothermic cardiac arrest may be acceptable for prolonged resuscitation time on CT imaging owing to reduced cerebral metabolism. These rare case reports demonstrate the potential benefits of CT imaging before ECPR in the management of hypothermic cardiac arrest with trauma and aid in appropriate candidate selection and effective intervention without compromising neurological outcomes.

## Introduction

Severe accidental hypothermia can cause circulatory instability and cardiac arrest [1]. Hypothermic cardiac arrest (HCA) is associated with a more favorable outcome than normothermic cardiac arrest owing to reduced oxygen demand and decreased cerebral metabolic rates [2]. Several studies demonstrated that extracorporeal cardiopulmonary resuscitation (ECPR) and cardiopulmonary bypass (CPB) lead to favorable outcomes in patients with HCA [3]. Conditions that generally preclude ECPR in normothermic cardiac arrest, such as unwitnessed arrest, asystole, pulseless electrical activity (PEA), and prolonged low- or no-flow time, are not absolute contraindications for ECPR in patients with HCA [4]. However, the outcome varies based on whether primary hypothermia causes the arrest or secondary hypothermia results from cardiac arrest. In particular, the coexistence of trauma and hypothermia in cardiac arrest cases poses a significant dilemma in the decision-making process for initiating extracorporeal life support (ECLS) rewarming. ECLS rewarming may be futile in cases where cardiac arrest results from severe head or torso injuries. In addition, ECLS can promote bleeding, which is further disadvantageous for patients with concomitant trauma. ECLS is an invasive procedure that requires significant resources; therefore, unnecessary initiation should be avoided. The Hypothermia Outcome Prediction after ECLS (HOPE) score [5], which is calculated by sex, age, the mechanism of hypothermia, the duration of cardiopulmonary resuscitation (CPR), serum potassium level, and core temperature at admission, may be used to predict survival in hypothermic cardiac arrest patients undergoing ECLS rewarming; however, it may not accurately predict outcomes in patients with trauma. Herein, we report two rare cases of cardiac arrest with concomitant trauma and hypothermia, in which ECPR was initiated after computed tomography (CT) imaging. Despite a slight prolongation in resuscitation time owing to CT imaging, this strategy facilitated appropriate case selection and resulted in successful resuscitation, demonstrating the broad resuscitation window advantage of HCA.

## Case presentation

Case 1

In 2022, a 74-year-old male collapsed on the ground in front of his home through an open second-floor window. The outside temperature on that day was -10°C, and the ground was covered with snow. Initial vital signs at the scene were a Glasgow Coma Scale (GCS) score of 13, a heart rate of 93 beats per minute (bpm), a blood pressure of 100/80 mmHg, and an axillary temperature of 25.7°C. The patient had split wounds on his forehead and bilateral knees with suspected fractures. In Japan, emergency medical teams are not equipped with rewarming devices except for blankets, so rewarming during transport was limited in efficiency. Upon arrival at the emergency department (ED), 40 minutes after being found, his cardiac rhythm changed to ventricular fibrillation, and cardiopulmonary resuscitation (CPR) was initiated. The patient received CPR with manual chest compressions and was intubated. The patient had a GCS score of 3 and a bladder temperature of 25.0°C. Blood gas analysis revealed a serum potassium level of 4.7 mmol/L. Although there was no evidence of intrathoracic or intra-abdominal hemorrhage on ultrasonography, non-contrast CT was performed before initiating extracorporeal membrane oxygenation (ECMO) owing to possible intracranial or retroperitoneal hemorrhage.

**Figure 1:**
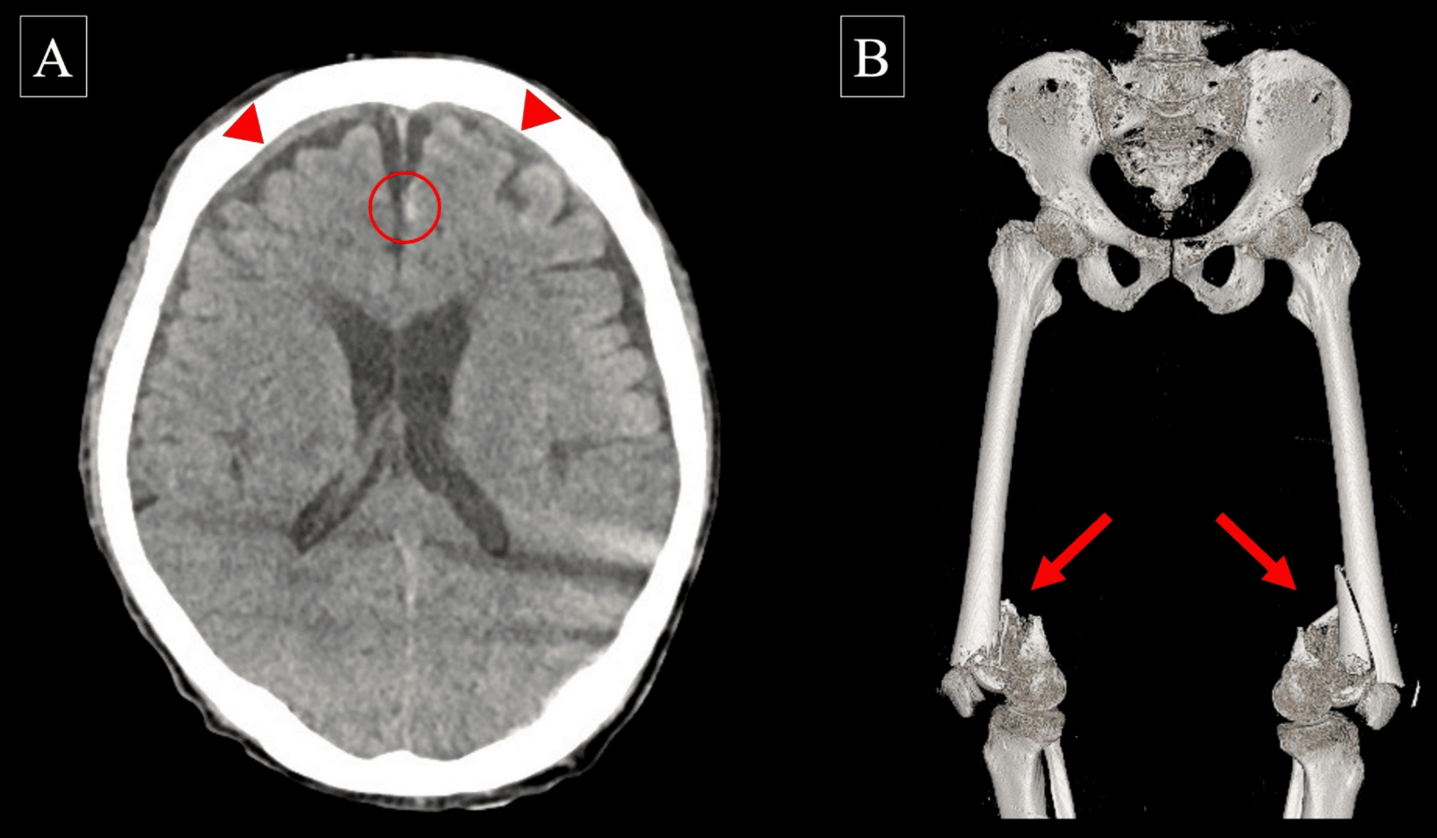
Head (A) and femur (B) CT images prior to the start of ECMO in case 1. The CT shows a traumatic subarachnoid hemorrhage (circle), acute subdural hematoma (arrowheads), and bilateral distal femur fractures (arrows). ECMO, extracorporeal membrane oxygenation; CT, computed tomography

**Figure 2:**
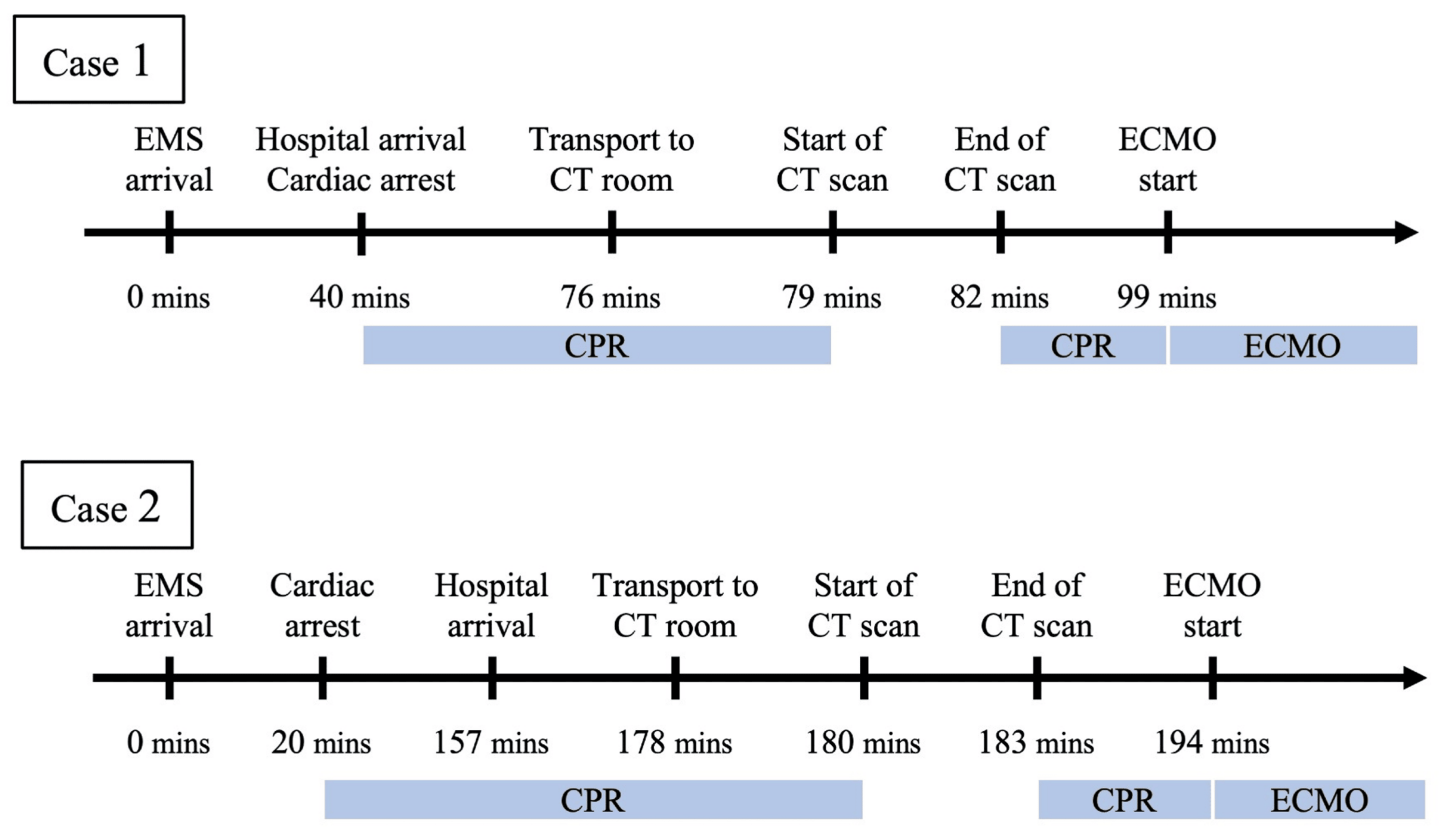
Clinical course. Case 1 was cardiopulmonary arrest immediately after hospital arrival. EMS, emergency medical service; CT, computed tomography; ECMO, extracorporeal membrane oxygenation; CPR, cardiopulmonary resuscitation

The CT scan, including transportation, took 16 minutes, with a three-minute interruption of CPR during imaging. Subsequently, the CT scan revealed a minor traumatic subarachnoid hemorrhage, acute subdural hematoma, and bilateral distal femur fractures, with no massive cerebral or torso hemorrhage (Figure 1). Hence, we performed ECLS rewarming without anticoagulants, and ECMO was initiated 59 minutes after cardiac arrest (Figure 2). We have added information to the case presentation indicating that when the bladder temperature of the patient reached 33°C, he reverted to sinus rhythm following a single 150 J biphasic defibrillation, with a gradual improvement in hemodynamics. After the external fixation of bilateral femoral fractures, the patient was admitted to the ICU. Follow-up CT showed a slight increase in the acute subdural hematoma; however, it could be treated conservatively with close monitoring and without surgical intervention. The patient was successfully weaned from ECMO 12 hours after initiation, regained consciousness on the second day, and was discharged from the ICU on the 13th day. Bilateral total knee arthroplasty was performed on the 15th day. After prolonged treatment for renal dysfunction and depression, he was transferred to a rehabilitation hospital 79 days after onset. At discharge, he had a Cerebral Performance Category (CPC) scale score of 1, indicating good cerebral performance, as the CPC scale ranges from 1 (good cerebral performance) to 5 (death). The HOPE survival probability was 37% when CT imaging was performed (calculated with a CPR duration of 59 minutes) and 43% when it was not performed (calculated with a CPR duration of 43 minutes).

Case 2

**Figure 3:**
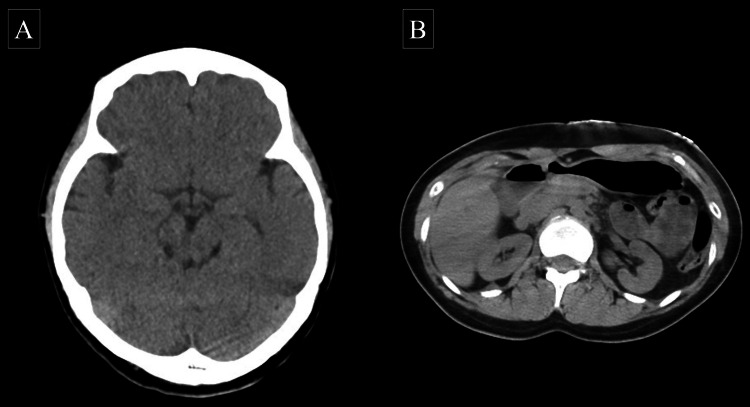
Head (A) and abdomen (B) CT images prior to the start of ECMO in case 2. The CT shows no trauma findings in the head and trunk. ECMO, extracorporeal membrane oxygenation; CT, computed tomography

In 2023, a 32-year-old female was observed jumping from a bridge that was 50 m high in a mountainous area on a snow-covered surface. Although she had slight spontaneous breathing and body movements, when the rescuers reached 145 minutes after the incident, CPR with manual chest compression was initiated following subsequent respiratory arrest during the rescue. On arrival at the hospital, her cardiac rhythm was asystole, and 137 minutes had passed since the cardiac arrest. She was immediately intubated upon arrival at the hospital. The patient had a GCS score of 3, dilated pupils with no light reflex, and a bladder temperature of 17.4°C. Blood gas analysis revealed a serum potassium level of 3.1 mmol/L. Physical examination and ultrasonography showed no evidence of trauma; however, non-contrast CT imaging was performed before initiating ECMO due to possible intracranial or retroperitoneal hemorrhage. The CT scan, including the time taken for transportation, took eight minutes, with a three-minute interruption of CPR during imaging. Subsequently, trauma findings and airway obstruction were absent on CT (Figure 3). Therefore, we performed ECLS rewarming without anticoagulants, and ECMO was initiated 174 minutes after the cardiac arrest (Figure 2). Twenty-four minutes after the initiation of ECMO, ventricular fibrillation developed when the bladder temperature reached 28°C. On reaching a bladder temperature of 31°C, the patient reverted to sinus rhythm after a single 150 J biphasic defibrillation. Subsequently, spontaneous respiration and light reflex recovered, and body movements appeared. The patient was successfully weaned off ECMO, regained consciousness on day 3, and was discharged from the ICU on day 7. At discharge 78 days after onset, the patient had a CPC scale score of 1. The HOPE survival probability was 95% both when CT imaging was performed (calculated with a CPR duration of 174 minutes) and when it was not performed (calculated with a CPR duration of 166 minutes).

## Discussion

In this study, we performed ECLS rewarming preceded by CT in patients with suspected HCA complicated by trauma and treated them safely without causing neurological deficits.

Trauma combined with hypothermia renders a poor prognosis, regardless of the presence of cardiac arrest [6]. In contrast, HCA often allows full recovery if there is no trauma or hypoxemia before hypothermia [1]. There have been reports of cases in which ECLS was applied after excluding major hemorrhage on echocardiography upon hospital arrival in patients with polytrauma and hypothermia who experienced cardiac arrest during transport, resulting in full neurological recovery [7]. ECLS may be effective even in cardiac arrest combined with hypothermia and trauma; however, severe traumatic brain injury and cardiac arrest preceding hypothermia must be ruled out. This decision may be difficult based solely on physical and echocardiography findings. Anticoagulation can be avoided in ECMO for patients with hypothermia or trauma, and it was not used in these two cases. However, it is important to note that ECMO-associated coagulopathy can still occur even without anticoagulation and may contribute to hemorrhage [8].

There have been several reports on the utility of CT after ECPR; however, reports on CT before ECPR are limited, and this is the first case involving hypothermia. In a retrospective study of patients with non-hypothermic PEA who were considered candidates for ECPR, a hybrid emergency room approach in which CT was performed before determining ECMO candidacy suggested that CT may be useful for decision-making [9]. CT performed before ECPR can detect cardiac arrest with poor neurological prognosis due to conditions such as brain hemorrhage and aortic dissection, thereby avoiding unnecessary ECMO. Even in patients with HCA and trauma, CT before ECLS rewarming may help avoid inappropriate ECLS applications.

However, performing and transporting CT can interrupt and extend CPR. Extensions in no-flow and low-flow durations are associated with poor prognosis in cardiogenic cardiac arrest [10,11]. In HCA, brain metabolism is significantly reduced, allowing for higher tolerance to interruptions in chest compressions and prolonged resuscitation time. Several case reports have documented successful full recovery following ECLS with intermittent CPR in patients with HCA. The European Resuscitation Council Guidelines allow for interruptions of less than five minutes in CPR for cardiac arrest patients with core temperatures below 28°C when continuous CPR is difficult and mechanical chest compression devices are unavailable [12,13]. The use of mechanical chest compression devices helps maintain CPR quality and reduce interruption times; however, it could cause artifacts in CT results, making accurate assessment challenging. In this report, CPR was interrupted for three minutes for CT imaging, and quality was reduced for several minutes during transport; however, it did not affect the neurological prognosis. The HOPE survival probability was calculated for each case, both with and without CT imaging, resulting in probabilities of 43% and 37% for case 1 and 95% and 95% for case 2. The loss of survival probability due to CT imaging was slight, and in both cases, the probabilities exceeded the 10% cutoff value considered when deciding to withhold ECLS rewarming [14]. The interruption and reduction in CPR quality due to the time required for CT imaging and transport appear to have a minimal impact on the neurological prognosis of patients with HCA. Additionally, in patients with HCA and trauma, CT imaging may provide crucial prognostic information that is not included in the HOPE score.

## Conclusions

In conclusion, our study highlights the potential benefits of performing CT imaging prior to initiating ECPR in patients experiencing hypothermic cardiac arrest with concomitant trauma. This approach, as demonstrated in our case reports, facilitates appropriate patient selection by identifying or ruling out significant traumatic injuries and ensuring that the cardiac arrest is due to hypothermia rather than other causes. Although the CT imaging process slightly prolongs the duration of CPR, our findings indicate that this might not adversely affect the neurological outcomes of patients, possibly due to the protective effects of reduced cerebral metabolism in hypothermic conditions. Both cases in our study showed full neurological recovery despite prolonged resuscitation times, underscoring the acceptability and potential advantages of this treatment strategy.
